# Painless Dilatation of Urethra

**Published:** 1874-01

**Authors:** 


*CHICAGO MEDICAL JOURNAL—December*, 1873 :

Painless Dilatation of the Urethra.—A new device for this purpose is mentioned in a French journal. It simply consists in the employment of a column of liquid about twenty metres high, established by means of a funnel, and containing about a pound and a half of water, (boiled at 25° or 27° C.) and suspended above the patient's bed. An india rubber tube—about two metres long—and provided with a cock in the middle of its length, so as to moderate or suspend the current of water, and having at its end a small glass pipe like an ordinary syringe, which is to be introduced into the meatus urinarius, connects the apparatus with the penis. The glass end being introduced, the cock is more or less opened at will, and slight pressure is exerted on the glans to prevent the water from running outside. The water in the funnel is then forced down by its own weight, and runs down drop by drop, dilating the stricture without pain, and, through its local and antiphlogistic action, rendering the urethra pervious to sounds and bougies. The patient can himself apply the apparatus three or four times a day, and when it is removed the surgeon has only to make use of his sounds or bougies.

